# Tropical cyclone-specific mortality risks and the periods of concern: A multicountry time-series study

**DOI:** 10.1371/journal.pmed.1004341

**Published:** 2024-01-22

**Authors:** Wenzhong Huang, Zhengyu Yang, Yiwen Zhang, Thomas Vogt, Ben Armstrong, Wenhua Yu, Rongbin Xu, Pei Yu, Yanming Liu, Antonio Gasparrini, Samuel Hundessa, Eric Lavigne, Tomas Molina, Tobias Geiger, Yue Leon Guo, Christian Otto, Simon Hales, Farnaz Pourzand, Shih-Chun Pan, Ke Ju, Elizabeth A. Ritchie, Shanshan Li, Yuming Guo, MCC Collaborators

**Affiliations:** 1 Climate, Air Quality Research Unit, School of Public Health and Preventive Medicine, Monash University, Melbourne, Australia; 2 Potsdam Institute for Climate Impact Research, Potsdam, Germany; 3 Department of Public Health Environments and Society, London School of Hygiene & Tropical Medicine, London, United Kingdom; 4 Centre on Climate Change & Planetary Health, London School of Hygiene & Tropical Medicine, London, United Kingdom; 5 Centre for Statistical Methodology, London School of Hygiene & Tropical Medicine, London, United Kingdom; 6 Environmental Health Science and Research Bureau, Health Canada, Ottawa, Canada; 7 School of Epidemiology and Public Health, University of Ottawa, Ottawa, Canada; 8 Department Applied Physics, Universitat de Barcelona, Barcelona, Spain; 9 Deutscher Wetterdienst (DWD), Regional Climate Office Potsdam, Potsdam, Germany; 10 Department of Environmental and Occupational Medicine, National Taiwan University (NTU) and NTU Hospital, Taipei, Taiwan; 11 National Institute of Environmental Health Sciences, National Health Research Institutes, Miaoli, Taiwan; 12 Institute of Environmental and Occupational Health Sciences, College of Public Health, National Taiwan University, Taipei, Taiwan; 13 Department of Public Health, University of Otago, Wellington, New Zealand; 14 School of Earth Atmosphere and Environment, Monash University, Melbourne, Australia; 15 Department of Civil Engineering, Monash University, Melbourne, Australia

## Abstract

**Background:**

More intense tropical cyclones (TCs) are expected in the future under a warming climate scenario, but little is known about their mortality effect pattern across countries and over decades. We aim to evaluate the TC-specific mortality risks, periods of concern (POC) and characterize the spatiotemporal pattern and exposure-response (ER) relationships on a multicountry scale.

**Methods and findings:**

Daily all-cause, cardiovascular, and respiratory mortality among the general population were collected from 494 locations in 18 countries or territories during 1980 to 2019. Daily TC exposures were defined when the maximum sustained windspeed associated with a TC was ≥34 knots using a parametric wind field model at a 0.5° × 0.5° resolution. We first estimated the TC-specific mortality risks and POC using an advanced flexible statistical framework of mixed Poisson model, accounting for the population changes, natural variation, seasonal and day of the week effects. Then, a mixed meta-regression model was used to pool the TC-specific mortality risks to estimate the overall and country-specific ER relationships of TC characteristics (windspeed, rainfall, and year) with mortality. Overall, 47.7 million all-cause, 15.5 million cardiovascular, and 4.9 million respiratory deaths and 382 TCs were included in our analyses. An overall average POC of around 20 days was observed for TC-related all-cause and cardiopulmonary mortality, with relatively longer POC for the United States of America, Brazil, and Taiwan (>30 days). The TC-specific relative risks (RR) varied substantially, ranging from 1.04 to 1.42, 1.07 to 1.77, and 1.12 to 1.92 among the top 100 TCs with highest RRs for all-cause, cardiovascular, and respiratory mortality, respectively. At country level, relatively higher TC-related mortality risks were observed in Guatemala, Brazil, and New Zealand for all-cause, cardiovascular, and respiratory mortality, respectively. We found an overall monotonically increasing and approximately linear ER curve of TC-related maximum sustained windspeed and cumulative rainfall with mortality, with heterogeneous patterns across countries and regions. The TC-related mortality risks were generally decreasing from 1980 to 2019, especially for the Philippines, Taiwan, and the USA, whereas potentially increasing trends in TC-related all-cause and cardiovascular mortality risks were observed for Japan.

**Conclusions:**

The TC mortality risks and POC varied greatly across TC events, locations, and countries. To minimize the TC-related health burdens, targeted strategies are particularly needed for different countries and regions, integrating epidemiological evidence on region-specific POC and ER curves that consider across-TC variability.

## Author summary

**Why was this study done?:**

- Tropical cyclones (TCs), among the most destructive and costliest climate extreme events, are expected to be more intense due to climate change.
- Despite the widely acknowledged hazards, a consistent and quantitative assessment of the mortality risks of TC across countries is lacking. Such quantitative and comparable evidence across countries is urgently required to better understand the health effects and respond to the potentially increasing hazards.
- No previous studies have characterized the periods of concern (POC), exposure-response (ER) relationship, and temporal trends of the TC health risks, directly relevant to more precise and effective preparedness and mitigation strategies.

**What did the researchers do and find?:**

- Using mortality data from 494 TC-exposed locations in 18 countries or territories, we quantified the TC-specific mortality risks and POC of the 382 TC events that affected these locations. The ER relationships and temporal trends were then characterized for each country or territory.
- TC exposure was associated with a prolonged elevated risk of all-cause, cardiovascular, and respiratory mortality, with an overall average POC of around 20 days.
- The TC mortality risks and POC varied greatly across TC events, locations, and countries.
- Overall, the mortality risks increased approximately linearly with increasing TC-related maximum sustained windspeed or cumulative rainfall.
- Most studied countries or territories witnessed a decreasing TC-related mortality risks from 1980 to 2019, especially for the Philippines, Taiwan, and the USA, while potentially increasing TC-related all-cause and cardiovascular mortality risks were observed for Japan.

**What do these findings mean?:**

- TC events can exhibit significant variations in their risk patterns, and future risk assessments may need to better account for this large across-TC variability.
- Targeted and evidence-based disaster management and preparedness strategies need to be developed for different countries to more effectively mitigate the TC hazards.
- Key study limitations include potential exposure misclassification errors, residual confounding, and limited generalizability.

## Introduction

Tropical cyclones (TCs), including hurricanes, typhoons, and tropical storms, dominate weather-related disaster damages [1] and pose a major threat to our society and health [2]. It has been estimated that TCs exposed 150 million people [3] and caused billions of US dollars in damages [4,5] annually worldwide. With continued growth in coastal populations and global warming, the impacts of TCs are expected to worsen due to the increasing exposed population and proportion of very intense TCs (e.g., the warmer surface ocean is likely fueling more powerful TCs with higher windspeed and precipitation) [6–8]. These indicate that TCs will likely remain an important public health concern. Quantifying their spatiotemporal health risks has important implications for understanding the health effects and helps develop strategies to mitigate and respond to the foreseen health burden.

Emerging evidence suggests an increased risk of adverse health outcomes, mostly all-cause hospitalizations or mortality, associated with TC exposure [9–16]. Except for the immediate physical impacts such as drowning and injuries, TCs also have been found to introduce persisting or delayed elevated mortality and morbidity risks, partially attributable to medical support disruptions, environmental contamination, and psychosocial stress [17]. These indirect and longer-term effects of TC could increase the cardiovascular and respiratory mortality and morbidity, which consist of a major and important part of the disease burden indirectly attributable to TCs. However, previous studies on TC epidemiology largely focused on a single TC event (mostly Hurricane Katrina, Sandy) restricted to a single year or area (mostly in the United States of America [USA]) [13] and focused on all-cause mortality. For example, 8 studies assessed the excess mortality in Puerto Rico after Hurricane Maria [18–25], but varied greatly in the estimated number of excess mortality (point estimates of all-cause excess mortality ranged from 514 to 4,645) due to the different utilized designs (e.g., various timeframes) and tools (e.g., survey versus mortality registration). Therefore, the results of single-TC studies may not be comparable and generalize well given the high heterogeneity in TC and population characteristics, study period/design, infrastructure, and modeling approaches across studies.

To compensate for the limitation on generalizability, several more recent studies have included multiple TCs spanning more than a decade and estimated the average health effects of TC exposures at county level in the USA [9–11,26]. While these studies revealed fundamental features of TC epidemiology in the USA, the multi-TC average health effects do not account for the across-TC variability [27]. The TC-specific health effects can vary greatly depending on the characteristics of the TC events and the population’s social structure and vulnerabilities. Additionally, very few studies have estimated the temporal trends in TC-related health risks, the exposure-response (ER) relationships, as well as the periods of concern (POC) of TCs, which were important aspects of strategic disaster management and resource allocation. For example, identifying patterns in the risk magnitude and the concerned periods after TCs with diverse characteristics across regions offers valuable evidence for efficiently allocating resources, optimizing preparedness efforts, and better understanding TC health effects. However, there is an overall knowledge gap in consistently exploring the spatiotemporal mortality risks associated with TCs across countries over a long timeframe.

To address these knowledge gaps, we aim to employ a recently proposed flexible statistical framework within the framework of a two-stage analysis based on a global dataset of multiple TCs and locations over long timeframes [24]. This advanced approach could account for the TC-specific POC and mortality risks, and has been shown higher accuracy and statistical power (i.e., stronger ability to detect small and persistent increases in mortality) compared to the Farrington model currently implemented by the US Center for Disease Control and Prevention (CDC) [24]. Specifically, we aim to consistently estimate the TC-specific POC and mortality risk across TCs, locations and countries, and characterize the spatiotemporal pattern of ER relationships of mortality risk with TC characteristics. Beyond all-cause mortality, we also included 2 other leading mortality outcomes, cardiovascular and respiratory mortality, to comprehensively capture and understand the health effects of TC, including the indirect effects that were largely unclear.

## Method

### Data collection

Based on the most updated Multi-Country Multi-City (MCC) Collaborative Research Network database, we integrated a global dataset of 1,914 locations from 44 countries or territories. Among these 1,914 locations, 494 locations from 18 countries or territories that experienced at least 1 TC during the data collection period were included in the study (**Table A in S2 Text**). The details of the MCC dataset have been described in our previous work [28,29].

Specifically, for each location in the MCC network, daily counts of all-cause mortality were collected and non-external causes (International Classification of Diseases [ICD], 9th Revision codes 0–799 or ICD-10 codes A0–R99) mortality were alternatively collected when all-cause mortality was unavailable. Two major and distinct causes of death, cardiovascular (ICD-10 codes I00–I99) and respiratory (ICD-10 codes J00–J99) mortality were also collected for each location. Cardiovascular and respiratory mortality, largely attributable to the indirect consequences of TCs, such as property loss, resource depletion, and disruptions in medical support, constitute a major part of the TC-associated mortality burden [17]. Except for the MCC data, we also collected data on all individual deaths (date, cause, and location of death) in Australia between 2009 and 2017 from the Australian Cause of Death Unit Record File [30], in New Zealand between 2000 and 2018 from the New Zealand Ministry of Health [31], in Brazil between 1996 and 2019 from the Brazil Mortality Information System (Sistema de Informação sobre Mortalidade, SIM) [32], and in Canada between 1986 and 2015 from the Vital Statistics Deaths Database of Statistics Canada [33]. For a time-series analysis, we aggregated these individual death data at location and daily level based on the administrative boundary with a proper area size for each country (Statistical Area Level 3 [SA3] for Australia [*n* = 316], territorial authority [TA] for New Zealand [*n* = 63], immediate region for Brazil [*n* = 510], and second-level administrative divisions [regions or districts within the provinces and territories] for Canada [*n* = 288]). MCC locations in Australia, New Zealand, Brazil, and Canada were thus excluded to avoid duplication. Consequently, the integrated global dataset covers 1,914 locations from 44 countries or territories, of which 494 locations from 18 countries or territories that experienced at least 1 TC during the data collection period were included. Among these 494 locations, the all-cause mortality data in 13 locations (2.6%) was represented by non-external mortality.

To estimate the annual population for each location, the annual gridded population per 10 years between 1980 and 2100 at a spatial resolution of 0.5° × 0.5° (about 55 km grid), derived by ensemble learning technique and models (R-squared values ranged from 0.81 to 0.84), was also collected from the Global Carbon Project [34]. The population data were first interpolated with a natural spline function of the available values to each year for each grid [29]. The annual population of each location was then calculated as the sum of the population of the grid cells in that year covered by that location.

### Exposure assessment

We used the improved Holland wind field model [35] to estimate the global historical temporal dynamics of the windspeed associated with TCs, which has been successfully applied in previous studies [8,36,37]. The methodology of this model has been described in detail in our previous work [3]. Briefly, we first obtained historical information on TCs including the position (i.e., center latitude and longitude coordinates), surface central pressure, radius, and the maximum sustained windspeed from the International Best Track Archive for Climate Stewardship (IBTrACS), a collection of best track data of TCs from sources worldwide [38]. The above variables served as inputs for the Holland wind field model as implemented within the CLIMADA Python package, an open-source impact modeling framework available on GitHub (https://github.com/CLIMADA-project/climada_python) [39]. We generated the daily wind profile (i.e., the grid-level daily 1-min sustained wind speeds associated with the cyclone) for each cyclone event in IBTrACS from January 1st, 1980 to December 31st, 2019, at a spatial resolution of 0.5° × 0.5°. The estimated global historical TC-related windspeed showed a good agreement in the validation analysis of reported wind fields in the regional dataset (Pearson correlation of r = 0.86). For each location, we defined TC exposure days as days with a TC-related maximum sustained wind speed ≥34 knots (17.5 m/s, 63 km/h, 39 mph; gale-force wind on the Beaufort scale) for the grid cell of the location [9,10]. For each TC in each location, we defined the TC hit day, t_0_, as the first day with a sustained wind speed ≥34 knots for that TC in that location. We obtained the cumulative rainfall (mm) at t_0_ for each location from the ERA-5 reanalysis data, which is created by assimilating historical weather data from numerous platforms (e.g., satellite, ground-based stations, radar, boats, airplanes, buoys) using sophisticated data assimilation models [40] and has been widely used in previous studies [41–45], as an additional metric of TC exposure besides the maximum sustained windspeed.

### Statistical analysis

We adopted a two-stage analytical approach to characterize the association between TC exposure and mortality. In the first stage, we implemented a flexible statistical framework that permitted both the surveillance of concerning increases in mortality rates and careful characterization of the effect of a past event to estimate the POC and excess mortality for each TC in each location [24,46,47]. The theoretical framework and methodological details have been well described elsewhere [24]. Briefly, the daily death counts were modeled with the following mixed Poisson model with a log-link:

$$Y_{it}|\varepsilon_{it}∼\mathrm{Poisson}(\mu_{it}[1+f_{i}(t)]\varepsilon_{it})$$ (1)

$${\mu_{it}=\mathrm{offset}(N}_{iy}{)e}^{\alpha_{i}(t)+s_{i}(t)+w_{i}(t)}$$ (2)

In the core formula of Eq (1), *Y*_*it*_ and *μ*_*it*_ represent the observed and expected deaths on day *t* in location *i*, respectively; (*t*) refers to the relative increase in mortality on day *t* in location *i* due to a TC and (*t*)*100 is the percent increase; and *ε*_*it*_ is a time series of auto-correlated random variables representing natural variability on day *t* in location *i*. The expected deaths, *μ*_*it*_, can be further decomposed according to Eq (2), where *N*_*iy*_ is the population on year *y* in location *i* and the log of population is treated as an offset to account for the change in population size; *α*_*i*_(*t*) is a smooth function of time that accounts for the secular changes (i.e., a slow-moving trend); and *w*_*i*_(*t*) and *s*_*i*_(*t*) are day-of-week effects and a yearly periodic function representing a seasonal trend, respectively. During the periods without TC exposure (i.e., the control periods), we assumed (*t*) = 0. When different from 0, (*t*) was assumed to be smooth enough to be represented by a smoothing cubic spline that provides enough flexibility to detect unusual mortality fluctuation due to an extreme event like a TC. To estimate the component of interest, (*t*), the *μ*_*it*_ and correlation structure of *ε*_*it*_ were first estimated based on the mortality during the control periods. Then, the *f*_*i*_(*t*) and the standard error (SE) were estimated using the Central Limit Theorem approximation that assumed $\hat{f_{i}}(t)$ followed a normal distribution and accounts for the uncertainty in the expected mortality rate. Using estimates of $\hat{f_{i}}(t)$ and the SE, the POC was defined as a post-TC period during which a percent increase of 0 is not in the 95% confidence interval (CI) for $\hat{f_{i}}(t)$ (i.e., the lower limit of the 95% CI of the estimated excess mortality is greater than 0).

We permitted a discontinuity on the TC hit day, t_0_, to account for a sudden direct effect and fitted a smoother spline, with 6 knots per year in the main model, to provide more power to detect subtle indirect effects [24]. If a location was exposed to multiple TCs during the data collection period, we excluded the two-month (60 days) post-TC periods to exclude the effects of other TCs. The final results for the first stage were presented as the POC and excess mortality (with 95% CI) for each TC in each location. The relative risk (RR) was also calculated as the observed deaths divided by the expected deaths (i.e., observed deaths minus excess deaths) for each TC. Sensitivity analyses by excluding a different length of post-TC period (30 days, 90 days) were conducted to test the robustness of the results.

In the second stage, with the TC-specific RRs (with 95% CI) from the first stage, we further used a mixed meta-regression model, accounting for the hierarchical structure of the RRs (a location could have several TC-specific RRs for multiple TCs, i.e., TCs nested within locations), to characterize the ER relationships of TC-related mortality risks with TC characteristics. Specifically, we built a univariate meta-regression model between the TC-specific RRs and the TC-related maximum sustained wind, cumulative rainfall, and the calendar year of TC hit, respectively, to examine the univariate ER relationship. All of these terms were included in the meta-regression models with a natural spline function of 2 degrees of freedom (df), as determined by a minimum Akaike information criterion (AIC), to allow for nonlinear ER relationships.

All data organization and analyses were conducted in R software, version 4.0.3 (Foundation for Statistical Computing, Vienna, Austria) [48]. The first- and second-stage analysis were conducted using the “excess_model” function from the “excessmort” package [49] and the “mixmeta” function from the “mixmeta” package [50], respectively.

## Results

### Deaths, TC, and periods of concern

**Fig 1:**
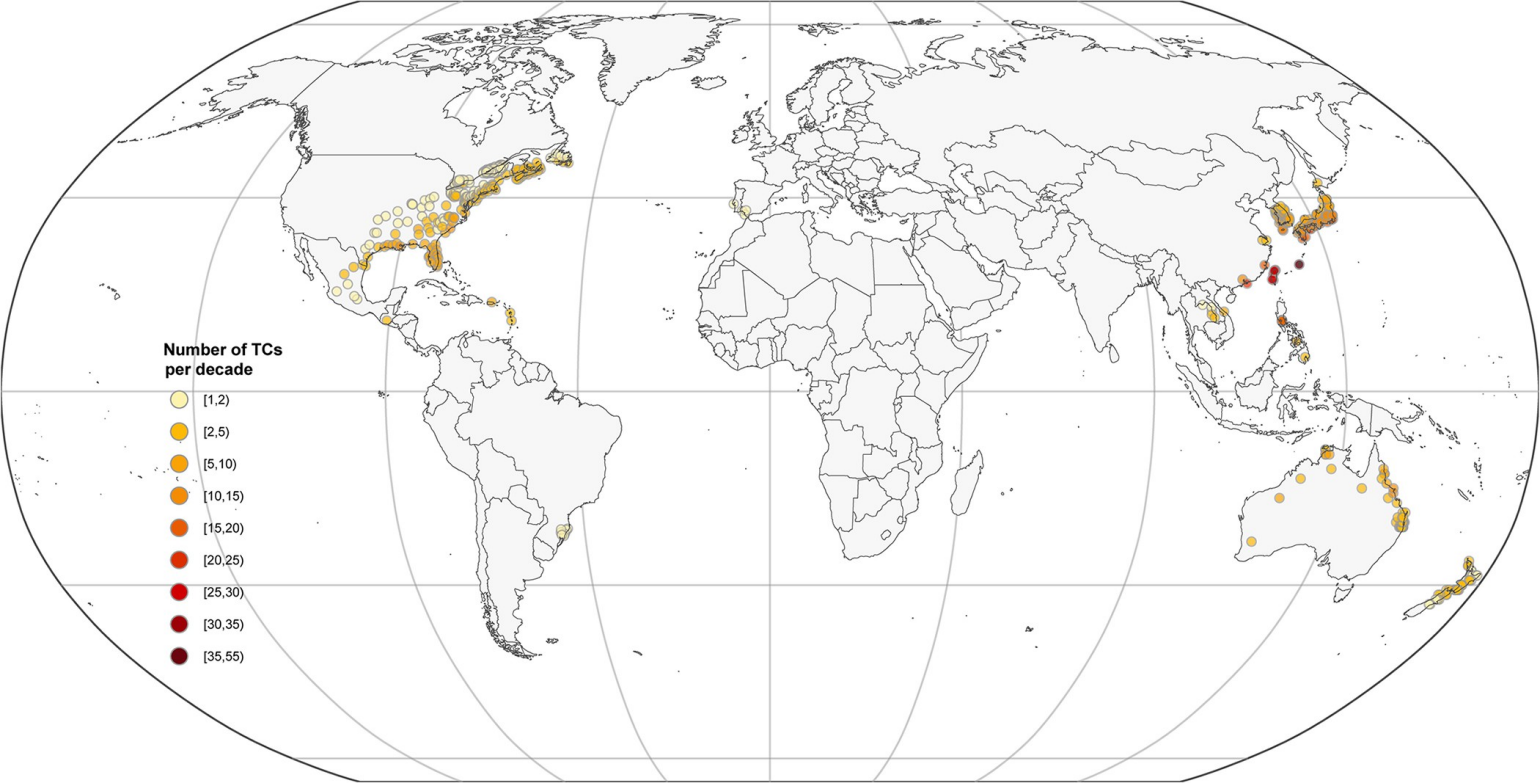
The spatial distribution and number of exposed TCs of the 494 study locations. . The base layer of the world map was imported from the public domain Natural Earth project (source: https://www.naturalearthdata.com/downloads/; terms of use: www.naturalearthdata.com/about/terms-of-use/). TC, tropical cyclone.

The spatial distribution of the included 494 locations and a summary of these locations (e.g., study periods, number of deaths, and TCs) are shown in **Fig 1** and **Table A in S2 Text**, respectively. In total, 47.7 million all-cause deaths, 15.5 million cardiovascular deaths, and 4.9 million respiratory deaths were included in the analyses. Each location contributed an average of 21 years (standard deviation [SD]: 9.4) of data. A total of 382 TC events that hit these 494 locations during the study period were included, with an average of 7 TC events for each location (**Fig 1**). The number of exposed TCs per decade varied substantially by location, ranging from 1 to 55. Locations in Taiwan (e.g., Taipei, Kaohsiung), Japan (e.g., Naha, Okinawa), the Philippines (e.g., Manila, Valenzuela) experienced TC most frequently (average number of TC per decade ≥14), whereas the lowest number (1–2) were mainly observed in locations from Brazil, New Zealand, and Canada. Overall, the average length of POC after a TC for all-cause mortality was 22 days (SD: 51.3), with great variations across and within countries (**Table A in S2 Text**). The average POC were relatively longer for the study locations in the USA, Brazil, and Taiwan (>30 days), while shorter for the study locations in Vietnam, Mexico, Australia, and New Zealand (≤10 days). Similar overall average POC and great variations across and within countries were also observed for TC-related cardiovascular and respiratory mortality (**Table A in S2 Text)**. The estimated overall and country-specific POC were robust to sensitivity analyses by excluding a different length of post-TC period (30, 60, and 90 days) (**Table B in S2 Text**).

### TC-related excess mortality and risk

**Fig 2:**
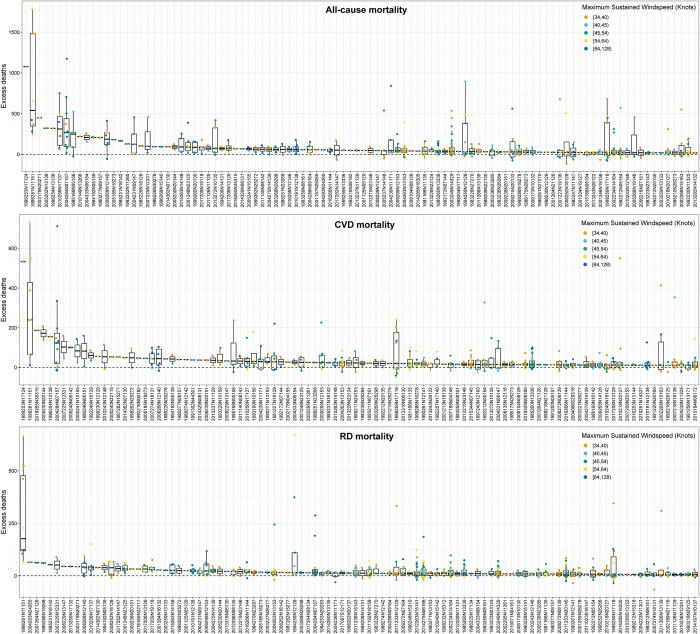
The top 100 TCs with highest excess deaths from all-cause, CVDs, and RDs. Each point in the figure indicates a location, and each tick on the X-axis represents a TC, which is identified by its IBTrACS event ID. A boxplot was fitted for the location-specific TC-related excess deaths within each TC. Each box represents the IQR of the excess deaths of each TC, with the middle bolded black line in the box representing the median value. The whiskers extending from the box indicate a range of 1.5 times the IQR. CVD, cardiovascular disease; IQR, interquartile range; RD, respiratory disease; TC, tropical cyclone.

**Fig 3:**
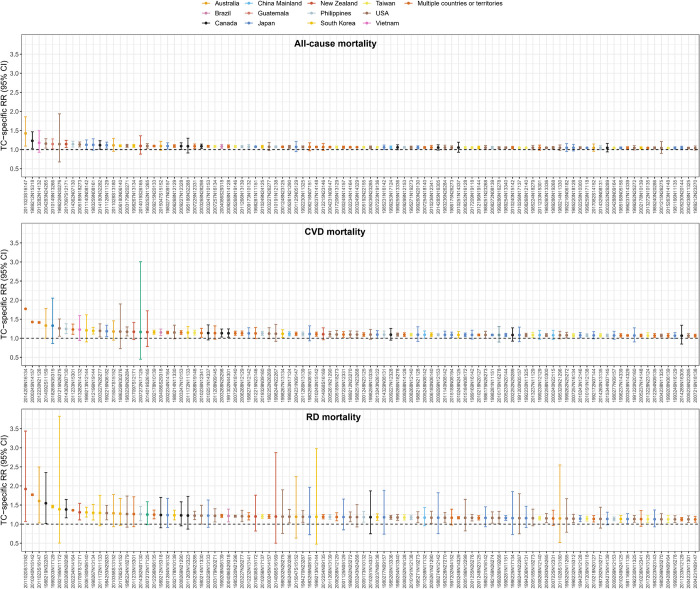
The top 100 TCs with highest RR for all-cause, CVDs, and RDs mortality. The RRs were estimated by comparing the deaths on TC-exposed days with those on non-exposed days, after adjusting for population changes, natural variation, seasonal, and day of the week effects. CVD, cardiovascular disease; RD, respiratory disease; RR, relative risk; TC, tropical cyclone.

**Fig 4:**
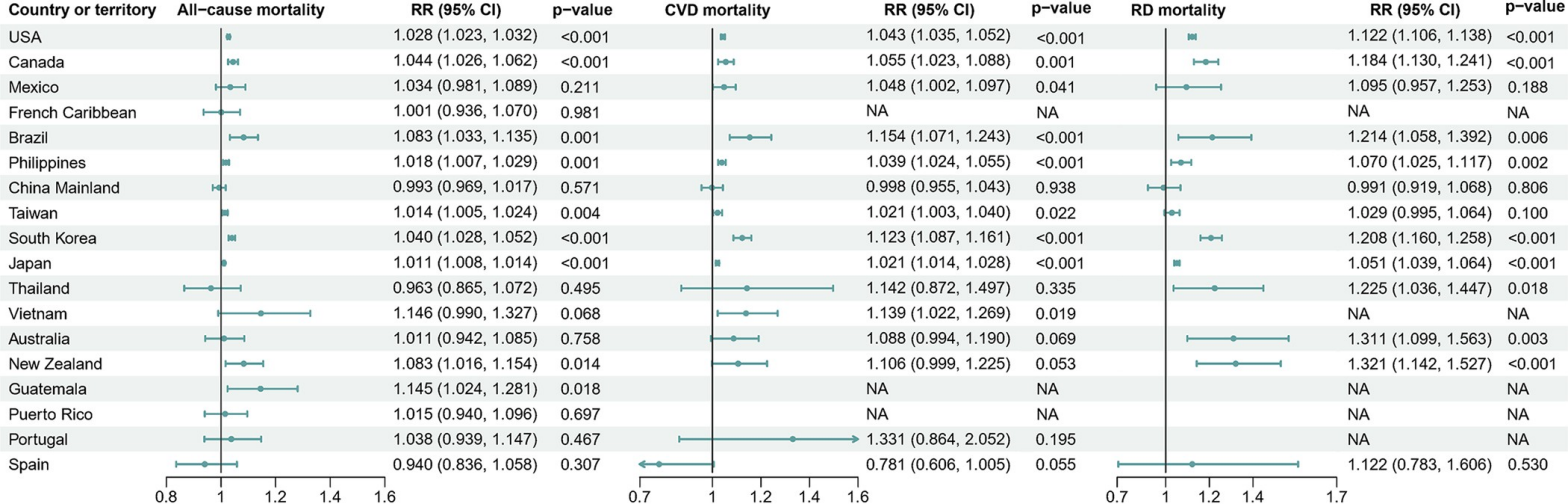
Country or territory-specific overall RR with 95% CI for all-cause, CVDs, and RDs mortality associated with TC exposure. The RRs indicated the mortality risks in TC days compared to non-TC days. CI, confidence interval; CVD, cardiovascular disease; RD, respiratory disease; RR, relative risk; TC, tropical cyclone.

The TC-location-specific excess mortality for the 100 TCs with the highest excess deaths is shown in **Fig 2**, with each point indicating a location and each tick on the x-axis representing a TC (a TC with a unique ID recorded in the IBTrACS). Large variations are observed for the TC-related excess deaths within and across TCs. For example, the TC of “1999253N17124” had the highest TC-related excess deaths, which hit 1 location (Hong Kong) in China Mainland in 1999 with a maximum sustained windspeed of 40 to 45 knots and caused around 1,076 deaths (**Fig 2**). Despite that the TC events that contributed most respiratory and cardiovascular deaths were different from those contributed most all-cause deaths, similar patterns of large inter- and intra-TC variability were also observed (**Fig 2**). The TC-specific RRs varied substantially, with a point estimate ranging from 1.04 to 1.42, 1.07 to 1.77, and 1.12 to 1.92 among the 100 TCs with highest RRs for all-cause, cardiovascular, and respiratory mortality, respectively (**Fig 3**). A maximum RR of 1.42 (95% CI [1.09, 1.86], *p* = 0.009), 1.77 (95% CI [1.76, 1.78], *p* < 0.001), and 1.92 (95% CI [1.07, 3.44], *p* = 0.028) for all-cause, cardiovascular, and respiratory mortality were observed for TC “2011023S16147” (Australia, 2011), “2014209N16134” (Japan and South Korea, 2014), and “2011020S13182” (New Zealand, 2011), respectively. At country level, relatively higher and statistically significant TC-related mortality risks were observed in Guatemala for all-cause mortality, Brazil, Vietnam, and South Korea for cardiovascular mortality, and New Zealand and Australia for respiratory mortality (**Fig 4**). The country-specific RR was generally robust to sensitivity analyses by excluding a different length of post-TC period (**Table C in S2 Text**).

### ER curve of TC-related windspeed with mortality by country or territories

**Fig 5:**
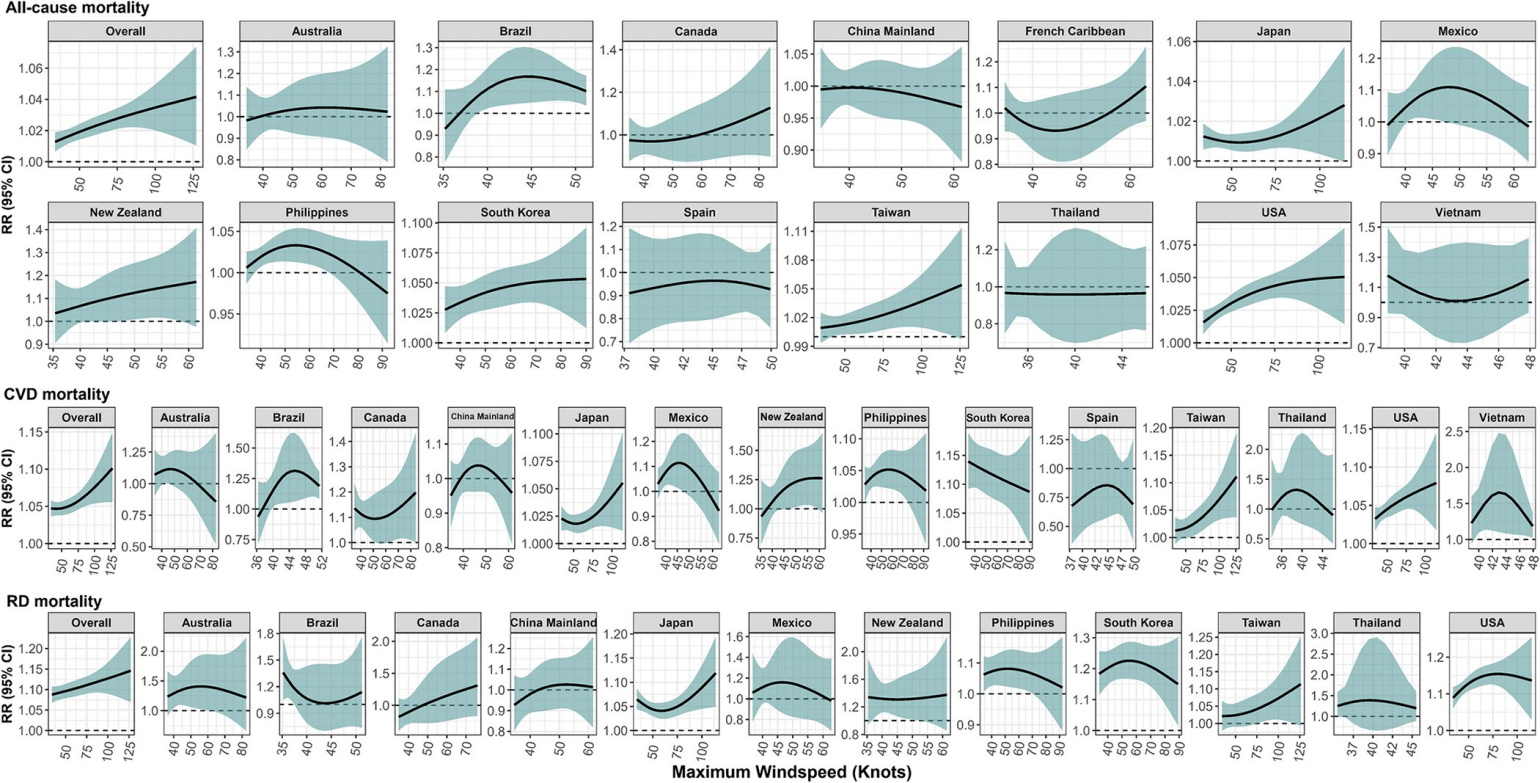
The exposure-response relationship of the RR for all-cause, CVDs, and RDs mortality with TC-related maximum sustained windspeed (knots) by countries or territories. The RRs indicated the mortality risks in TC days compared to non-TC days. CVD, cardiovascular disease; RD, respiratory disease; RR, relative risk; TC, tropical cyclone.

When characterizing the associations of TC-related maximum sustained windspeed with mortality risk, we observed an overall monotonically increasing and non-threshold curve with approximately linear shape for the ER relationships for all-cause, cardiovascular, and respiratory mortality (**Fig 5**). At country level, we found generally significant, positive linear or supra-linear ER curves of TC-related maximum sustained windspeed with mortality in Japan, South Korea, Taiwan, and the USA for all-cause mortality; Japan, Taiwan, and the USA for cardiovascular mortality; and Japan, Taiwan, and the USA for respiratory mortality (**Fig 5**). The positive ER curves were consistently observed for Japan, Taiwan, and the USA. However, there is insufficient evidence to support significant and positive ER curves between TC-related maximum sustained windspeed and mortality in other countries or regions including China Mainland, Mexico, and Thailand (**Fig 5**). Sensitivity analysis by excluding a different length of post-TC period showed robust overall and country-specific ER relationships of TC-related maximum sustained windspeed with mortality, with a similar shape to that in the main model (**Fig A in S2 Text**).

### ER curve of TC-related precipitation with mortality by country or territories

We observed similar patterns for the overall ER relationships of TC-related cumulative rainfall with mortality, with consistently significant and positive linear curves for all-cause, cardiovascular, and respiratory mortality (**Fig B in S2 Text**). Positive linear or supra-linear and monotonically increasing ER curves were detected in Japan, the Philippines, South Korea, Taiwan, and the USA for all-cause mortality; the Philippines, Taiwan, and the USA for cardiovascular mortality; and Taiwan and the USA for respiratory mortality. Consistent monotonically increasing ER curves were consistently observed for Taiwan and the USA. No sufficient evidence of a significant and positive ER curve between TC-related cumulative rainfall and mortality in other countries or regions including China Mainland, Vietnam, Mexico, and Thailand. The estimated overall and country-specific ER relationships were robust to sensitivity analyses by excluding a different length of post-TC period (**Fig C in S2 Text**).

### Temporal trends of TC-related mortality by country or territories

**Fig 6:**
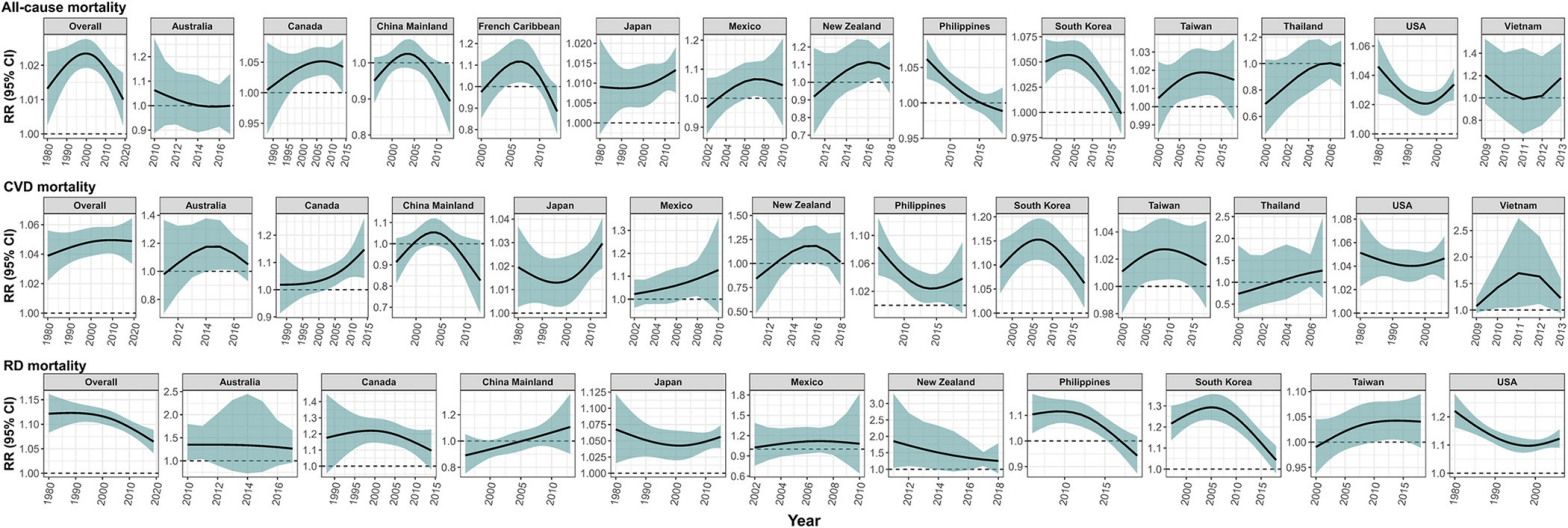
The temporal trends of the RR for all-cause, CVDs, and RDs mortality by countries or territories from 1980 to 2019. The RRs indicated the mortality risks in TC days compared to non-TC days. CVD, cardiovascular disease; RD, respiratory disease; RR, relative risk; TC, tropical cyclone.

When considering the temporal variations of the TC-related mortality, an overall decreasing trend was found for all-cause and respiratory mortality, while not for cardiovascular mortality (**Fig 6**). At country level, an overall slightly increasing trend in TC-related all-cause mortality risk was observed in Japan and Taiwan, while decreasing trends in the Philippines, South Korea, and the USA; an increasing trend in TC-related cardiovascular mortality risk was observed in Japan and Mexico, while overall decreasing trends were observed in the Philippines, South Korea, and the USA; a decreasing increasing trend in TC-related respiratory mortality risk was observed in Japan, New Zealand, the Philippines, and the USA, while a potentially increasing trend was found for Taiwan (**Fig 6**). For the remaining countries including Australia, Canada and Vietnam, Thailand, Mexico and New Zealand, no sufficient evidence to detect a temporal trend. Generally, similar overall and country-specific temporal trends were observed in sensitivity analyses by excluding a different length of post-TC period (**Fig D in S2 Text**).

## Discussion

Our large-scale population-based study estimated the TC-specific POC and mortality risks, with substantial variations in TC-related mortality risk within and across TCs. Additionally, we characterized the ER relationships and found an overall monotonically increasing, non-threshold and approximately linear curve of TC-related maximum sustained windspeed and cumulative rainfall with all-cause and cardiopulmonary mortality risks. An overall decreasing trend was observed for TC-related all-cause and respiratory mortality risk from 1980 to 2019. Further heterogeneous patterns of the ER relationships and temporal trends were revealed at the country level, such as the increasing trend in TC-related all-cause mortality risk in Japan, yet a decreasing trend in the Philippines, South Korea, and the USA.

As expected, we found that the TC-related mortality risks varied considerably across and within TCs, evidencing the necessity to account for the across-TC variability when assessing the health effects of TCs. However, among the limited epidemiological studies that systematically assessed the health effects of multiple TC events, most estimated the multi-TC average effects [9–11,26,27]. These studies also utilized a wind field model to quantify the TC exposure for a large number of TCs and found an elevated risk of mortality [9,27], hospitalization [10,11], and preterm birth [26] associated with TCs in the USA for the past decades. To our knowledge, only 1 study has estimated the TC-specific health risks, which included all TCs in the USA from 1999 to 2015 and also observed large variations in the TC-related excess mortality across and within TCs [27]. However, this study did not estimate the TC-specific POC, but instead used an 11-day post-TC period as a hypothetical POC and calculated the excess deaths within this period for all TCs. TCs could impact public health through both direct and indirect pathways. Direct impacts including physical trauma (e.g., injury and drowning) during exposure could be more immediate, while indirect impacts such as the socio-psycho environmental stress, poor or mal-nutrition due to TCs (e.g., loss of property and resources, evacuation, interruption of medical and social support) could manifest at a longer-term to increase the mortality and morbidity. Therefore, a POC of several days may not be able to sufficiently capture the health impacts of TCs, especially for the indirect impacts, as indicated by a POC of more than 1 month for some countries/TCs in our results. Additionally, TCs could vary substantially in terms of their POC due to their different physical characteristics (e.g., windspeed, rainfall, and duration), as well as the population vulnerability. Such variability was reflected in our findings of a large standard deviation of the estimated POC across TCs, even within the same country. The self-specified identical exposure window (i.e., hypothetical POC) commonly used in previous multi-TC studies may introduce potential exposure misclassification and not be able to well capture the TC-related health effects.

To our knowledge, this study is the first to examine the ER relationships between TC characteristics and risk of mortality on a multicountry and multi-TC scale. Based on the estimated mortality risk and characteristics for each TC, we characterized the overall and country-specific ER curves of TC-related maximum sustained windspeed and cumulative rainfall with different mortality outcomes. We observed an overall monotonically increasing, non-threshold and approximately linear curve of TC-related maximum sustained windspeed and cumulative rainfall with all-cause, cardiovascular, and respiratory mortality risks. Only 1 prior study has estimated the ER curves of TC characteristics with health outcomes and observed a similar monotonically increasing ER relationship with an approximately linear shape between TC-related maximum sustained windspeed and all-cause mortality, hospitalizations for respiratory diseases, chronic obstructive pulmonary diseases, and cardiovascular diseases in the USA [27]. Stronger TCs with higher windspeed and rainfall were more likely to induce adverse events such as flood, displacement and power outage, there having higher risks causing mortality and morbidity. Prior epidemiological studies on multiple TCs generally assessed the potential hazard of TCs only based on a binary variable of exposure (exposed versus unexposed) in terms of the maximum sustained windspeed [9–11,26,27,41,51]. The established ER curves with TC-related windspeed and rainfall in the current study could inform the potential risks of various mortality outcomes associated with different TC intensities. Considering that more intense TCs are expected in the future under a changing climate, it is critical to incorporate the epidemiological evidence such as the ER curves in the early warning system to accurately evaluate the potential hazards of a landfalling TC and develop strategies accordingly to minimize the health burdens [52].

Heterogeneous TC-related mortality risks and patterns of the ER relationships across countries and mortality outcomes were further revealed in our study. Populations in countries like Guatemala, Brazil, Vietnam, and South Korea appear to be especially vulnerable to TC-related elevated mortality risks compared to those in other countries or regions such as Canada, Mexico, and Australia. Many factors, such as topography, economics, disaster management practices, and population characteristics, can impact the susceptibility to natural disasters like TCs [53–55]. However, there is very little evidence on systematic assessment of the vulnerability to TCs across countries and no clear explanation for such differences has yet been proposed. The higher susceptibility to the elevated mortality risks of TCs in these regions could be partially attributed to the higher frequency of high-amplitude TCs, while the relatively fewer strong TCs or study locations in other countries including Mainland China and Mexico hindered us from detecting a significant and positive ER curve [56–59]. Additionally, Clark and colleagues proposed a Notre Dame Global Adaptation Index (ND-GAIN) and attributed the differences in the overall vulnerability to climate change across countries to 6 country-level life-supporting sectors—food, water, health, ecosystem service, human habitat, and infrastructure [60]. Countries with more reliable water and food supply (e.g., higher fresh water withdrawal rate), better health and ecosystem services (e.g., less slum population and dependency on natural capital), and improved infrastructure and human habitat (e.g., less population living under 5 m above sea level and smaller age dependency ratio) could be more resilient to the adverse impacts of climate change. The heterogeneous patterns of the ER relationships could also be explained by differences in these factors across countries. Overall, current epidemiological evidence on the potential contributing factors or effect modifiers on the associations between TCs and health is still very limited and inconclusive. More studies are required to better elucidate this issue and the underlying contributors.

We observed an overall decreasing trend for TC-related all-cause and respiratory mortality risks, with great heterogeneity across countries or territories. Despite the temporal change of the TC characteristics (e.g., tracks, frequency, intensity, duration) has been well characterized [56–59,61,62], no studies have yet estimated the temporal trend of TC-related mortality risks. To our knowledge, only 1 study examined the temporal change in the risk of homelessness, casualties, and property losses induced by TCs in South Korea and found a decreasing trend over 1979 to 2010, which is similar to our observed ER curve for TC-related all-cause mortality in South Korea [63]. Additionally, the mostly decreasing TC-related mortality risk across countries highlights the effectiveness and progress of the disaster management measures and devoted prevention efforts, especially for the Philippines, Taiwan, and the USA. While the intensity and duration of landfall TCs have been increasing [64,65], the improved early warning system and disaster preparedness practices can significantly reduce the related health risks [66–68]. However, it should also be noted that a potentially increasing trend in TC-related all-cause and cardiovascular mortality risk was observed in Japan. This may be partially attributed to the considerably increasing proportion of the elderly population and the prevalence of cardiovascular diseases over the past decade in this country [69,70]. Further studies are highly warranted to elucidate the underlying mechanisms and formulate targeted approaches to reverse the increasing trends.

This study had 4 main strengths. To the best of our knowledge, this is the first and largest global investigation of the mortality risk attributed to TCs. Compared with most previous studies confined to single or several TC events within a limited region or timeframe, we collected representative death data from countries or territories of the USA (including the locations from the territories in the Caribbean [i.e., Virgin Islands and Puerto Rico]), Japan, South Korea, Canada, Brazil, Taiwan, Australia, and New Zealand. We also developed TC exposure data based on a collection of representative and best track data of TCs from official sources worldwide (i.e., IBTrACS), which has been widely used to analyze TC ecology and subsequent events (e.g., flood) [62,71–73]. A final of 382 TC events in 494 locations from 18 countries or territories during 1980 to 2019, which were characterized by different climates, socioeconomics, demographics, public health service development, and TC features. This allowed us to characterize the spatiotemporal pattern of the TC-related mortality risks, and to reduce potential selection-related biases and ensure the high-quality and generalizability of our findings. Compared to the Farrington Model currently implemented by the CDC for evaluating related disaster-attributable deaths [74–76], we employed an advanced flexible statistical framework that has higher power to detect the small and persistent increases in death rate introduced by such effects as one contiguous period [24]. Additionally, this modeling technique enabled us to account for the great across-TC variability and evaluated TC-specific POC and mortality risks, which tends to provide more precise effect estimates than the traditional multi-TC average estimates based on an identical exposure window. Finally, apart from providing the TC-related mortality risk estimates like in most prior studies, we further estimated the POC, the ER curves between TC characteristics and mortality outcomes, as well as the temporal trends of mortality risks, which were important aspects for developing or adjusting disaster management policies and public health interventions to mitigate the adverse impacts of TCs.

There are also some limitations in our study. The possibility of residual confounding cannot be completely excluded. Despite our application of an advanced methodology to first attempt to estimate the TC-specific POC across countries, some uncertainty in the estimates caused by the limitations of this methodology must be acknowledged. We involved a predetermined wash-out period by excluding a certain length of post-TC period for other TCs (if any) when estimating the individual mortality risks for each TC event. The impacts of other TCs may still exist and bias our results. However, we believed the impact of this issue could be modest as evidenced by the robust results in sensitivity analyses by excluding different lengths of post-TC period. Furthermore, the determination of POC relied on statistical significance (i.e., the lower limit of the 95% CI > 0), potentially influenced by factors irrelevant to TCs, such as sample size. However, a series of simulation studies indicated that the impact of this issue on results may be modest, with consistent and reliable estimates in different scenarios [24]. Nonetheless, more studies are needed in the future to work on this challenging topic and improve the estimates of POC across TCs. In addition, mandatory pre-TC evacuation orders and population displacement during TC could be important influential factors in the health impacts of TCs. However, to our knowledge such data have never been systematically compiled and available on a multi-TC scale [24]. To minimize the health threats of climate and weather-related disasters, it is highly warranted to collect, compile, and incorporate richer data on these events in future studies. In this study, we estimated the yearly population size for each location and included it as an offset in the model to account for the long-term variations over time and across space due to the unavailability of daily population size in each location. Population characteristics such as the age and sex structure were associated with the vulnerability to TC hazards. Based on a classical two-stage modeling approach, we accounted for the temporal variations within a location by the control of temporal trends in the first stage, and the spatial variations across locations by using a mixed meta-regression model in the second stage. However, due to the lack of these data (e.g., age- or sex-specific daily mortality) at daily level and on a multicountry scale, we were thus unable to assess the potential risk differences across subpopulations such as different age and sex subgroups. Moreover, the TC-specific excess deaths for some TCs could be underestimated for some countries or territories without nationwide data such as China Mainland, Vietnam, and Mexico. The limited locations in China Mainland, Vietnam, and Mexico also increased the uncertainty of our results and prevented us from detecting significant findings for those countries and regions. We were also not able to estimate the TC-related mortality risk in the countries or territories without study locations but with a potentially high TC-related health burden (e.g., Bangladesh, Myanmar). These issues warrant further exploration with more comprehensive data and should be lessened in the future as the MCC network expands. Finally, land conditions could significantly influence the speed and direction of surface winds. The widely used improved wind field model by Holland incorporated an attenuation factor, the ratio between the distance to the center and the radius of maximum winds, to resemble surface friction effects [8,35–37]. This does not explicitly account for the surface friction-induced wind speed reduction [77] or motion-induced asymmetry [78]. Post-landfall, TC wind fields could become very noisy due to interaction with complex land surfaces, posing challenges in accounting for uncertainties when assessing the ER relationship with health outcomes [79]. However, neglecting inhomogeneous wind conditions over land is expected to minimally impact the results, given the study’s focus on a binary TC exposure variable (exposed versus unexposed).

## Conclusion

To conclude, TCs show great variation in the POC and elevated mortality risks globally. The overall ER relationships of TC-related windspeed and rainfall with all-cause and cardiopulmonary mortality exhibited a monotonically increasing, non-threshold and linear curve, with a heterogeneous pattern across regions. An overall decreasing trend was observed for the TC-related all-cause and cardiovascular mortality risk from 1980 to 2019. The TC-related mortality risks were generally decreasing in most of the study countries, especially for the Philippines and the USA, while potentially increasing trends in TC-related all-cause and cardiovascular mortality risks were observed for Japan. Further targeted actions and in-depth explorations of TC epidemiology in the countries with high and increasing TC-related mortality burdens are particularly needed.

## Supporting information

S1 Text MCC Collaborators. (DOCX)

S2 Text Supplementary Material. (DOCX)

## Abbreviations

AIC: Akaike information criterion
CDC: Center for Disease Control and Prevention
CI: confidence interval
df: degrees of freedom
ER: exposure-response
IBTrACS: International Best Track Archive for Climate Stewardship
ICD: International Classification of Diseases
IDI: Integrated Data Infrastructure
MCC: Multi-Country Multi-City
ND-GAIN: Notre Dame Global Adaptation Index
POC: periods of concern
RR: relative risk
SA3: Statistical Area Level 3
SD: standard deviation
SIM: Sistema de Informação sobre Mortalidade
SE: standard error
TA: territorial authority
TC: tropical cyclone
USA: United States of America
