# Supplementary material for: Tropical cyclone-specific mortality risks and the periods of concern: A multicountry time-series study
Source: PLoS Med. 2024 Jan 22;21(1):e1004341. doi: 10.1371/journal.pmed.1004341 (PMC10843109; doi:10.1371/journal.pmed.1004341)
Supplement: S1 Text — (DOCX) [file pmed.1004341.s001.docx]

**Supporting Information**

**MCC Collaborators**

Dominic Roye PhD^1,2^, Mathilde Pascal PhD^3^, Paulo H. N. Sadiva^4^, Micheline S. Z. S. Coelho^4^, Prof. Shilu Tong PhD^5,6,7^, Pierre Masselot PhD^8,9^, Ana Maria Vicedo-Cabrera PhD^10,11,12^, Prof. Joel Schwartz PhD^13^, Prof. Haidong Kan PhD^14^, Prof. Patrick Goodman PhD^15^, Ariana Zeka PhD^16^, Prof. Masahiro Hashizume PhD^17^, Prof. Magali Hurtado Diaz PhD^18^, César De la Cruz Valencia MSc^18^, Xerxes Seposo PhD^19^, Baltazar Nunes PhD^20,21^, Joana Madureira PhD^22,23,24^, Prof. Ho Kim PhD^25^, Whanhee Lee PhD^26,27^, Aurelio Tobias PhD^28,19^, Carmen Íñiguez PhD^29,2^, Antonella Zanobetti PhD^13^, Tran Ngoc Dang PhD^30,31^, Do Van Dung PhD^31^

^1^Climate Research Foundation (FIC), Madrid, Spain, ^2^CIBER of Epidemiology and Public Health, Madrid, Spain, ^3^Department of Environmental and Occupational Health, Santé publique France, ^4^Department of Pathology, Faculty of Medicine, University of São Paulo, São Paulo, Brazil, ^5^School of Public Health and Social Work, Queensland University of Technology, Brisbane, Australia, ^6^School of Public Health and Institute of Environment and Human Health, Anhui Medical University, Hefei, China, ^7^Shanghai Children’s Medical Centre, Shanghai Jiao-Tong University, Shanghai, China, ^8^Department of Public Health Environments and Society, London School of Hygiene & Tropical Medicine, London, United Kingdom, ^9^Centre on Climate Change & Planetary Health, London School of Hygiene & Tropical Medicine, London, United Kingdom, ^10^Institute of Social and Preventive Medicine, University of Bern, Bern, Switzerland, ^11^Oeschger Center for Climate Change Research, University of Bern, Bern, Switzerland, ^12^Department of Public Health Environments and Society, London School of Hygiene and Tropical Medicine, London, United Kingdom, ^13^Department of Environmental Health, Harvard T.H. Chan School of Public Health, Boston, MA, USA, ^14^Department of Environmental Health, School of Public Health, Fudan University, Shanghai, China, ^15^School of Physics, Technological University Dublin, Dublin, Ireland, ^16^Institute for Environment, Health and Societies, Brunel University London, London, UK, ^17^Department of Global Health Policy, Graduate School of Medicine, The University of Tokyo, Tokyo, Japan, ^18^Department of Environmental Health, National Institute of Public Health, Cuernavaca, Morelos, Mexico, ^19^School of Tropical Medicine and Global Health, Nagasaki University, Nagasaki, Japan, ^20^Department of Epidemiology, Instituto Nacional de Saúde Dr Ricardo Jorge, Porto, Portugal, ^21^Centro de Investigação em Saúde Pública, Escola Nacional de Saúde Pública, Universidade NOVA de Lisboa, Lisbon, Portugal, ^22^Environmental Health Department, Instituto Nacional de Saúde Dr Ricardo Jorge, Porto, Portugal, ^23^EPIUnit-Instituto de Saúde Pública, Universidade do Porto, Porto, Portugal, ^24^Laboratório para a Investigação Integrativa e Translacional em Saúde Populacional (ITR), Porto, Portugal, ^25^Graduate School of Public Health, Seoul National University, Seoul, Republic of Korea, ^26^School of the Environment, Yale University, New Haven CT, USA, ^27^Department of Occupational and Environmental Medicine, School of Medicine, Ewha Womans University, Seoul, South Korea, ^28^Institute of Environmental Assessment and Water Research, Spanish Council for Scientific Research, Barcelona, Spain, ^29^Department of Statistics and Computational Research. Universitat de València, València, Spain, ^30^Institute of Research and Development, Duy Tan University, Da Nang, Vietnam, ^31^Department of Environmental Health, Faculty of Public Health, University of Medicine and Pharmacy at Ho Chi Minh City, Ho Chi Minh City, Vietnam
