# Supplementary material for: Tropical cyclone-specific mortality risks and the periods of concern: A multicountry time-series study
Source: PLoS Med. 2024 Jan 22;21(1):e1004341. doi: 10.1371/journal.pmed.1004341 (PMC10843109; doi:10.1371/journal.pmed.1004341)
Supplement: S2 Text — (DOCX) [file pmed.1004341.s002.docx]

# Supplementary Material

**Table of Contents**

| **Table A** | Summary of the study periods, number of locations, deaths, TC numbers and POC of the 494 study locations by countries or territories. |
| --- | --- |
| **Table B** | The estimated POC of tropical cyclones for all-cause, CVD and RD mortality by excluding a different length of post-TC period (30, 60, 90 days). |
| **Table C** | The country or territory-specific overall RR (with 95% CI) associated with TC for all-cause, CVD and RD mortality by excluding a 30-, 60- or 90-day post-TC period. |
| **Fig A** | The exposure-response (ER) relationship of the relative risks (RR) for all-cause, cardiovascular disease (CVD) and respiratory disease (RD) mortality with tropical cyclone (TC)-related maximum sustained windspeed (knots) by countries or territories. The ER relationships were fitted using TC-specific RRs estimated by excluding a 30-, 60- or 90-day post-TC period. |
| **Fig B** | The exposure-response relationship of the relative risks (RR) for all-cause, cardiovascular disease (CVD) and respiratory disease (RD) mortality with TC-related cumulative rainfall (mm) by countries or territories. |
| **Fig C** | The exposure-response (ER) relationship of the relative risks (RR) for all-cause, cardiovascular disease (CVD) and respiratory disease (RD) mortality with tropical cyclone (TC)-related cumulative rainfall (mm) by countries or territories. The ER relationships were fitted using TC-specific RRs estimated by excluding a 30-, 60- or 90-day post-TC period. |
| **Fig D** | The temporal trend of the relative risks (RR) for all-cause, cardiovascular diseases (CVD) and respiratory diseases (RD) mortality after TC exposure from 1980 to 2019 by countries or territories. The temporal trends were fitted using TC-specific RRs estimated by excluding a 30-, 60- or 90-day post-TC period. |

**Table A.** Summary of the study periods, number of locations, deaths, TC numbers and POC of the 494 study locations by countries or territories.

|  | Period | No. of locations | All-cause mortality | | CVD mortality | | RD mortality | |
| --- | --- | --- | --- | --- | --- | --- | --- | --- |
|  |  |  | No. of deaths | Average length of POC (SD)^a^ | No. of deaths | Average length of POC (SD)^a^ | No. of deaths | Average length of POC (SD)^a^ |
| **North America** |  |  |  |  |  |  |  |  |
| USA | 1980-2006 | 123 | 18621976 | 30 (58.1) | 6719977 | 22 (48.7) | 1632564 | 25 (54.4) |
| Canada | 1986-2015 | 104 | 3677546 | 20 (46.8) | 1236246 | 15 (40) | 314418 | 33 (58.4) |
| **South America** |  |  |  |  |  |  |  |  |
| Brazil | 1996-2019 | 8 | 286407 | 40 (51.8) | 88366 | 47 (61.1) | 32466 | 48 (75.3) |
| **Latin America and the Caribbean** |  |  |  |  |  |  |  |  |
| Guatemala | 2009-2016 | 1 | 62715 | NA | NA | NA | NA | NA |
| Mexico | 1998-2014 | 6 | 2551522 | 5 (11.6) | 660859 | 24 (32.8) | 244682 | NA |
| Puerto Rico | 2009-2016 | 1 | 26564 | NA | NA | NA | NA | NA |
| French Caribbean | 2000-2015 | 2 | 46190 | 15 (27.8) | NA | NA | NA | NA |
| **Eastern Asia** |  |  |  |  |  |  |  |  |
| China Mainland | 1996-2015 | 5 | 674842 | 29 (60.9) | 234830 | 27 (63) | 104144 | 24 (64.8) |
| Japan | 1980-2015 | 62 | 14589419 | 16 (47.1) | 4670454 | 17 (46.6) | 1973044 | 10 (35.7) |
| South Korea | 1997-2018 | 36 | 3070357 | 21 (48.8) | 701638 | 18 (44.9) | 222314 | 30 (61.6) |
| Taiwan | 2000-2018 | 6 | 1740776 | 33 (58.3) | 373288 | 21 (46.2) | 183139 | 26 (50.7) |
| **South-eastern Asia** |  |  |  |  |  |  |  |  |
| Philippines | 2006-2019 | 12 | 796933 | 20 (45.6) | 288555 | 26 (52.4) | 116027 | 27 (57.3) |
| Thailand | 1999-2008 | 5 | 102304 | NA | 14724 | NA | 9600 | NA |
| Vietnam | 2009-2013 | 1 | 6214 | 9 (15.6) | 2215 | 53 (85) | 166 | NA |
| **Southern Europe** |  |  |  |  |  |  |  |  |
| Portugal | 1980-2018 | 1 | 802500 | NA | 319657 | NA | 71547 | NA |
| Spain | 1990-2014 | 3 | 199468 | NA | 80111 | NA | 18720 | NA |
| **Australia and New Zealand** |  |  |  |  |  |  |  |  |
| Australia | 2009-2017 | 79 | 241352 | 5 (13.3) | 71174 | 26 (58.9) | 20436 | 17 (47.4) |
| New Zealand | 2000-2018 | 39 | 244586 | 10 (30) | 86373 | 20 (60.3) | 21468 | 49 (61.8) |
| **Overall** |  | 494 | 47741671 | 22 (51.3) | 15548467 | 20 (47.4) | 4964735 | 20 (49.6) |

Abbreviations: CVD, cardiovascular diseases; NA, not available/applicable; POC, periods of concern; RD, respiratory diseases; SD, standard deviation; TC, tropical cyclone.

^a^Due to the limited number of TCs during the study period or the unavailability of cardiovascular or respiratory diseases mortality data, the POC distribution for some countries or regions could not be calculated and is therefore shown as "NA".

**Table B.** The estimated POC^a^ of tropical cyclones for all-cause, CVD and RD mortality by excluding a different length of post-TC period^b^ (30, 60, 90 days).

| Country or territories | All-cause mortality | | | CVD mortality | | | RD mortality | | |
| --- | --- | --- | --- | --- | --- | --- | --- | --- | --- |
|  | 30 days | 60 days | 90 days | 30 days | 60 days | 90 days | 30 days | 60 days | 90 days |
| Australia | 4 (11.7) | 5 (13.3) | 5 (14.1) | 25 (58.7) | 26 (58.9) | 26 (59.1) | 13 (40.3) | 17 (47.4) | 13 (39.3) |
| Brazil | 41 (51.7) | 40 (51.8) | 42 (52.7) | 47 (61.1) | 47 (61.1) | 48 (61.9) | 47 (75.1) | 48 (75.3) | 49 (75.5) |
| Canada | 20 (46.9) | 20 (46.8) | 20 (47.1) | 14 (39.7) | 15 (40.0) | 15 (40.0) | 35 (58.1) | 33 (58.4) | 33 (58.8) |
| China Mainland | 32 (60.7) | 29 (60.9) | 33 (61.6) | 27 (63.0) | 27 (63.0) | 20 (55.4) | 22 (63.4) | 24 (64.8) | 28 (66.3) |
| French Caribbean | 14 (27.2) | 15 (27.8) | 15 (29.0) | NA | NA | NA | NA | NA | NA |
| Guatemala | NA | NA | NA | NA | NA | NA | NA | NA | NA |
| Japan | 16 (47.1) | 16 (47.1) | 17 (47.6) | 17 (46.3) | 17 (46.6) | 18 (46.4) | 10 (35.4) | 10 (35.7) | 11 (36.6) |
| Mexico | 5 (11.2) | 5 (11.6) | 5 (11.6) | 23 (31.9) | 24 (32.8) | 25 (34.3) | NA | NA | NA |
| New Zealand | NA | 10 (30.0) | 11 (32.0) | 20 (59.7) | 20 (60.3) | 20 (60.0) | 51 (70.7) | 49 (61.8) | 61 (64.5) |
| Philippines | 20 (46.7) | 20 (45.6) | 21 (45.1) | 27 (53.3) | 26 (52.4) | 28 (51.3) | 27 (57.6) | 27 (57.3) | 27 (57.1) |
| Portugal | NA | NA | NA | NA | NA | NA | NA | NA | NA |
| Puerto Rico | NA | NA | NA | NA | NA | NA | NA | NA | NA |
| South Korea | 21 (49.0) | 21 (48.8) | 22 (50.1) | 16 (40.9) | 18 (44.9) | 18 (41.9) | 30 (61.7) | 30 (61.6) | 30 (61.7) |
| Spain | NA | NA | NA | NA | NA | NA | NA | NA | NA |
| Taiwan | 35 (61.9) | 33 (58.3) | 36 (56.9) | 21 (49.4) | 21 (46.2) | 23 (47.8) | 28 (56.6) | 26 (50.7) | 31 (57.0) |
| Thailand | NA | NA | NA | NA | NA | NA | NA | NA | NA |
| USA | 30 (58.1) | 30 (58.1) | 29 (57.1) | 22 (49.1) | 22 (48.7) | 22 (49.1) | 26 (55.2) | 25 (54.4) | 25 (53.8) |
| Vietnam | 9 (15.6) | 9 (15.6) | 8 (13.9) | 53 (83.0) | 53 (85.0) | 52 (87.2) | NA | NA | NA |
| **Overall** | 22 (51.8) | 22 (51.3) | 23 (51.2) | 19 (47.2) | 20 (47.4) | 20 (46.9) | 20 (50.4) | 20 (49.6) | 21 (50.4) |

Abbreviations: CVD, Cardiovascular disease; NA, not applicable; POC, periods of concern; RD, respiratory diseases; SD, standard deviation.

^a^Due to the limited number of TCs during the study period or the unavailability of CVD or RD mortality data, the POC distribution for some countries or regions could not be calculated and is therefore shown as "NA".

^b^To exclude the effects of other TCs (if any) when estimated the POC for the TC of interest, a certain length of post-TC periods was excluded for other TCs.

**Table C.** The country or territory-specific overall RR^a^ (with 95% CI) associated with TC for all-cause, CVD and RD mortality by excluding a 30-, 60- or 90-day post-TC period^b^.

| Country or territories | All-cause mortality | | | CVD mortality | | | RD mortality | | |
| --- | --- | --- | --- | --- | --- | --- | --- | --- | --- |
|  | 30 days | 60 days | 90 days | 30 days | 60 days | 90 days | 30 days | 60 days | 90 days |
| USA | 1.028 (1.023, 1.032) | 1.028 (1.023, 1.032) | 1.027 (1.022, 1.032) | 1.043 (1.035, 1.051) | 1.043 (1.035, 1.052) | 1.044 (1.036, 1.053) | 1.125 (1.110, 1.139) | 1.122 (1.106, 1.138) | 1.121 (1.106, 1.136) |
| Canada | 1.044 (1.027, 1.062) | 1.044 (1.026, 1.062) | 1.045 (1.028, 1.063) | 1.053 (1.021, 1.085) | 1.055 (1.023, 1.088) | 1.092 (1.032, 1.155) | 1.228 (1.162, 1.297) | 1.184 (1.130, 1.241) | 1.189 (1.135, 1.246) |
| Mexico | 1.033 (0.981, 1.088) | 1.034 (0.981, 1.089) | 1.034 (0.981, 1.090) | 1.046 (0.999, 1.094) | 1.048 (1.002, 1.097) | 1.051 (1.007, 1.096) | 1.092 (0.954, 1.251) | 1.095 (0.957, 1.253) | 1.096 (0.958, 1.255) |
| French Caribbean | 1.000 (0.936, 1.068) | 1.001 (0.936, 1.070) | 1.004 (0.938, 1.075) | NA | NA | NA | NA | NA | NA |
| Brazil | 1.082 (1.032, 1.135) | 1.083 (1.033, 1.135) | 1.086 (1.036, 1.139) | 1.153 (1.071, 1.242) | 1.154 (1.071, 1.243) | 1.160 (1.077, 1.250) | 1.211 (1.052, 1.393) | 1.214 (1.058, 1.392) | 1.222 (1.067, 1.400) |
| Philippines | 1.019 (1.008, 1.029) | 1.018 (1.007, 1.029) | 1.021 (1.010, 1.032) | 1.038 (1.023, 1.053) | 1.039 (1.024, 1.055) | 1.044 (1.029, 1.060) | 1.070 (1.031, 1.110) | 1.070 (1.025, 1.117) | 1.070 (1.026, 1.116) |
| China Mainland | 0.999 (0.978, 1.021) | 0.993 (0.969, 1.017) | 1.000 (0.972, 1.029) | 0.996 (0.956, 1.038) | 0.998 (0.955, 1.043) | 1.005 (0.958, 1.055) | 1.011 (0.940, 1.088) | 0.991 (0.919, 1.068) | 0.999 (0.835, 1.196) |
| Taiwan | 1.013 (1.006, 1.021) | 1.014 (1.005, 1.024) | 1.018 (1.006, 1.029) | 1.020 (1.006, 1.034) | 1.021 (1.003, 1.040) | 1.024 (1.005, 1.043) | 1.030 (1.005, 1.055) | 1.029 (0.995, 1.064) | 1.041 (1.004, 1.079) |
| South Korea | 1.038 (1.026, 1.050) | 1.040 (1.028, 1.052) | 1.043 (1.030, 1.055) | 1.102 (1.073, 1.132) | 1.123 (1.087, 1.161) | 1.114 (1.084, 1.145) | 1.193 (1.147, 1.241) | 1.208 (1.160, 1.258) | 1.191 (1.150, 1.234) |
| Japan | 1.010 (1.007, 1.013) | 1.011 (1.008, 1.014) | 1.010 (1.006, 1.013) | 1.019 (1.013, 1.026) | 1.021 (1.014, 1.028) | 1.021 (1.010, 1.031) | 1.037 (1.021, 1.052) | 1.051 (1.039, 1.064) | 1.054 (1.041, 1.068) |
| Thailand | 0.964 (0.867, 1.073) | 0.963 (0.865, 1.072) | 0.963 (0.865, 1.072) | 1.140 (0.869, 1.496) | 1.142 (0.872, 1.497) | 1.146 (0.875, 1.501) | 1.223 (1.036, 1.443) | 1.225 (1.036, 1.447) | 1.225 (1.037, 1.448) |
| Vietnam | 1.164 (1.006, 1.347) | 1.146 (0.990, 1.327) | 1.110 (0.957, 1.288) | 1.208 (1.033, 1.414) | 1.139 (1.022, 1.269) | 1.112 (0.999, 1.239) | NA | NA | NA |
| Australia | 1.008 (0.940, 1.080) | 1.011 (0.942, 1.085) | 1.012 (0.943, 1.086) | 1.083 (0.989, 1.186) | 1.088 (0.994, 1.190) | 1.093 (1.000, 1.195) | 1.307 (1.110, 1.540) | 1.311 (1.099, 1.563) | 1.309 (1.113, 1.539) |
| New Zealand | 1.061 (0.985, 1.144) | 1.083 (1.016, 1.154) | 1.088 (1.022, 1.159) | 1.106 (0.998, 1.226) | 1.106 (0.999, 1.225) | 1.109 (1.001, 1.228) | 1.292 (1.107, 1.508) | 1.321 (1.142, 1.527) | 1.291 (1.274, 1.308) |
| Guatemala | 1.136 (1.008, 1.281) | 1.145 (1.024, 1.281) | 1.158 (1.063, 1.261) | NA | NA | NA | NA | NA | NA |
| Puerto Rico | 1.012 (0.937, 1.092) | 1.015 (0.940, 1.096) | 1.014 (0.940, 1.095) | NA | NA | NA | NA | NA | NA |
| Portugal | 1.041 (0.942, 1.150) | 1.038 (0.939, 1.147) | 1.036 (0.937, 1.145) | 1.323 (0.858, 2.041) | 1.331 (0.864, 2.052) | 1.337 (0.868, 2.062) | NA | NA | NA |
| Spain | 0.941 (0.836, 1.059) | 0.940 (0.836, 1.058) | 0.939 (0.835, 1.057) | 0.780 (0.605, 1.006) | 0.781 (0.606, 1.005) | 0.779 (0.605, 1.004) | 1.120 (0.782, 1.605) | 1.122 (0.783, 1.606) | 1.123 (0.785, 1.608) |

Abbreviations: CVD, Cardiovascular disease; CI, confidence interval; NA, not applicable; RD, respiratory diseases; RR, relative risk; SD, standard deviation.

^a^Due to the limited number of TCs during the study period or the unavailability of CVD or RD mortality data, the RR for some countries or regions could not be estimated and is therefore shown as "NA".

^b^To exclude the effects of other TCs (if any) when estimated the POC for the TC of interest, a certain length of post-TC periods was excluded for other TCs.


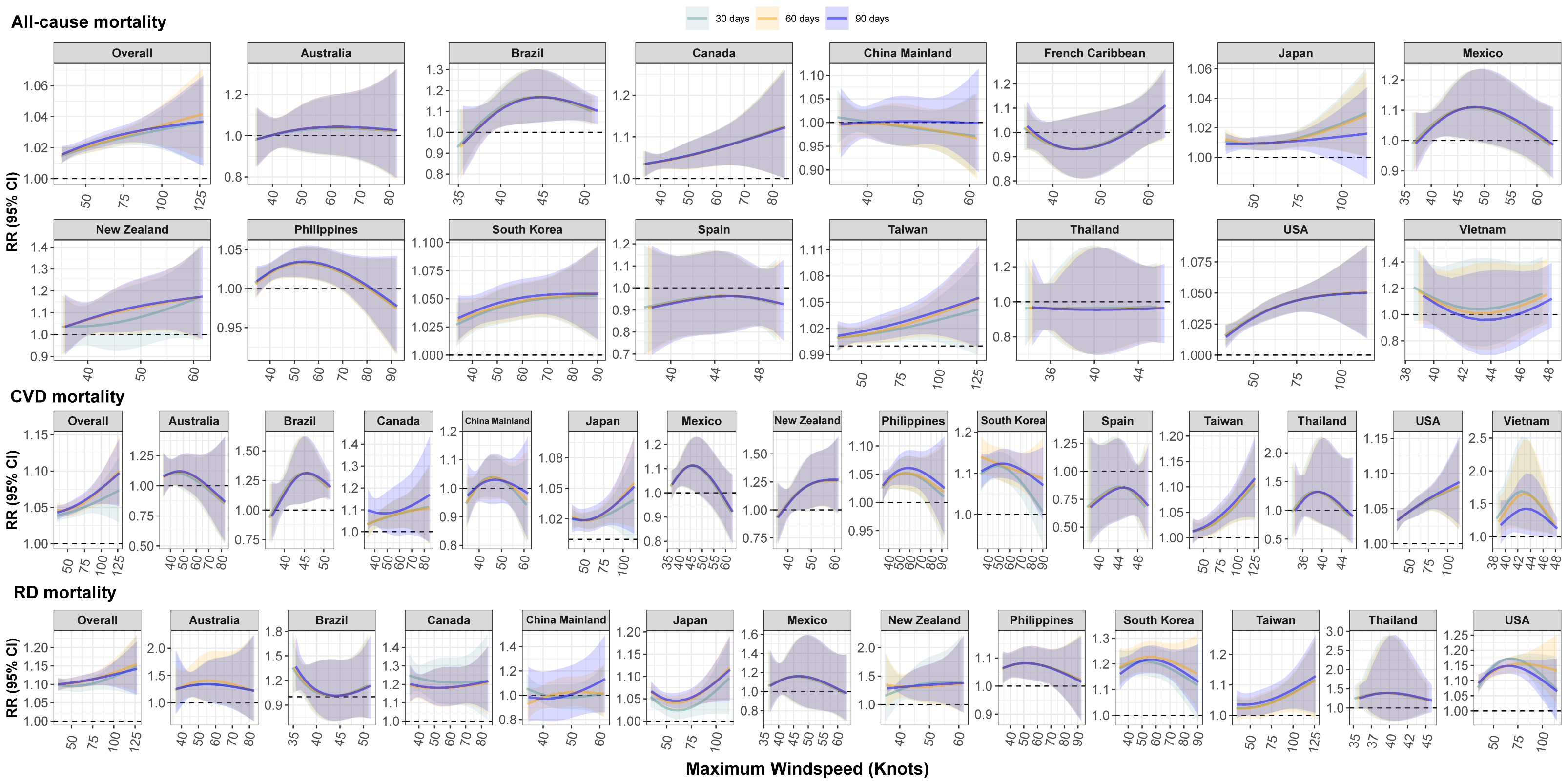


**Fig A**. The exposure-response (ER) relationship of the relative risks (RR) for all-cause, cardiovascular disease (CVD) and respiratory disease (RD) mortality with tropical cyclone (TC)-related maximum sustained windspeed (knots) by countries or territories. The ER relationships were fitted using TC-specific RRs estimated by excluding a 30-, 60- or 90-day post-TC period.


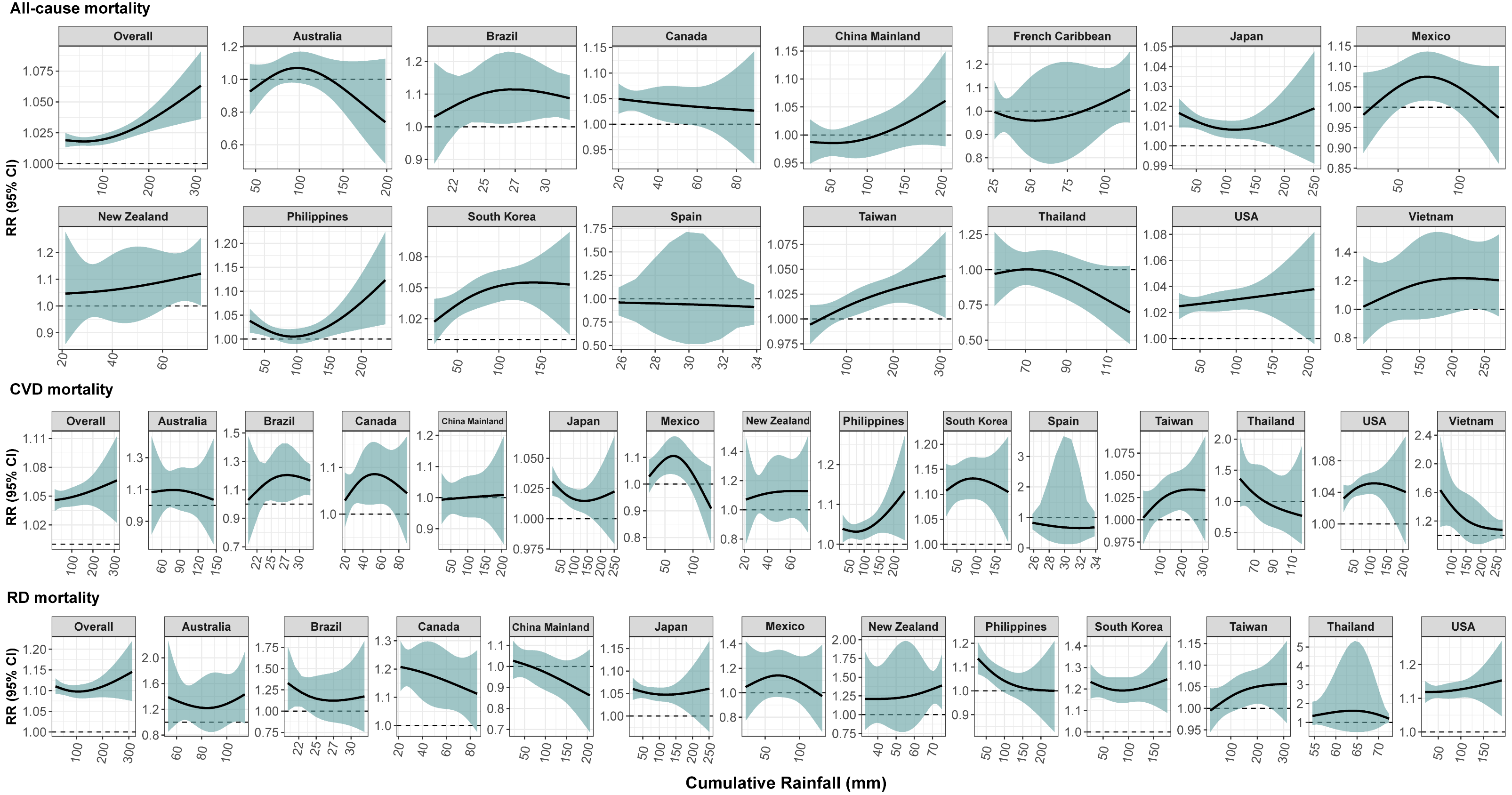


**Fig B.** The exposure-response relationship of the relative risks (RR) for all-cause, cardiovascular disease (CVD) and respiratory disease (RD) mortality with TC-related cumulative rainfall (mm) by countries or territories.


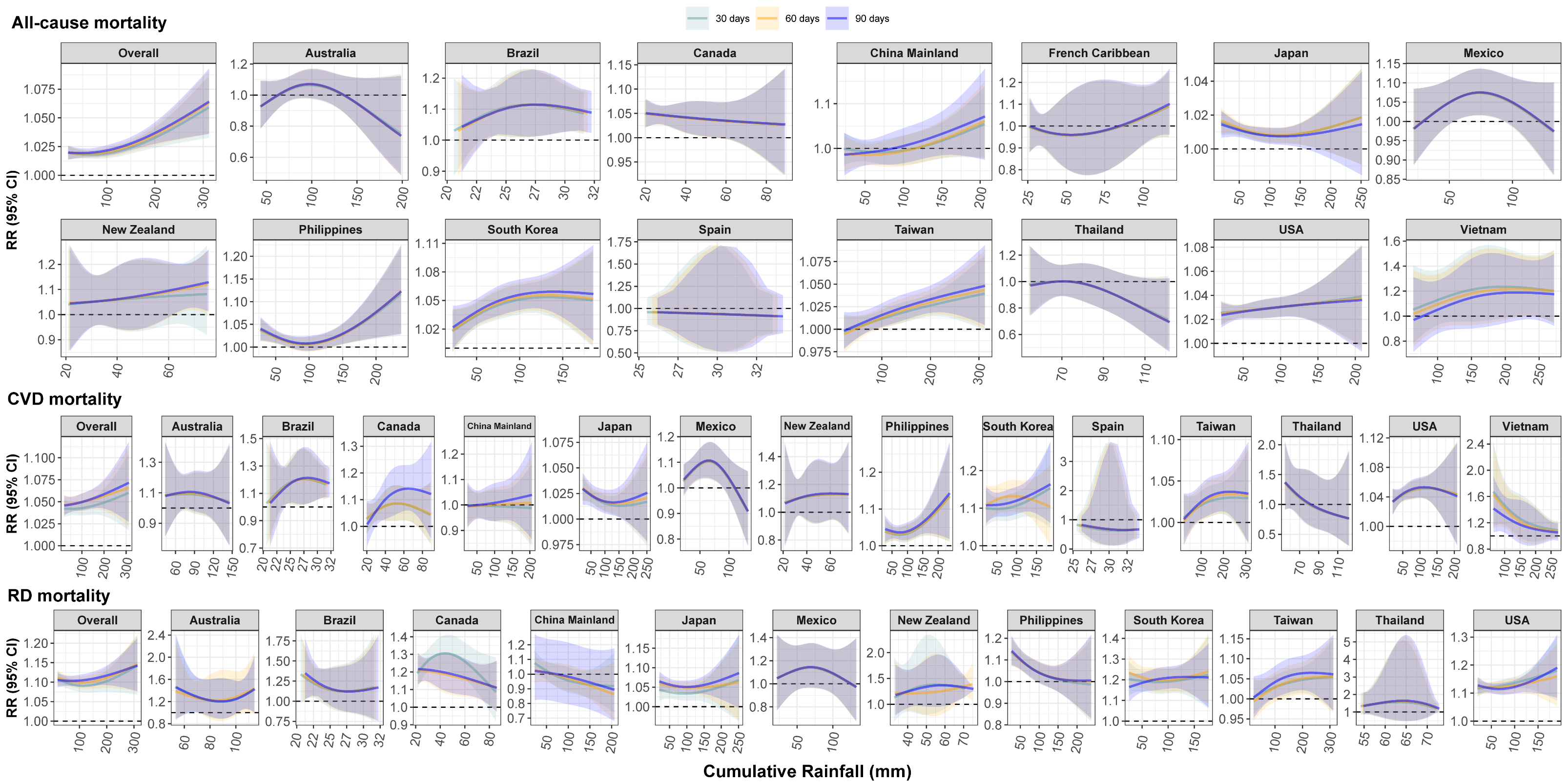


**Fig C**. The exposure-response (ER) relationship of the relative risks (RR) for all-cause, cardiovascular disease (CVD) and respiratory disease (RD) mortality with tropical cyclone (TC)-related cumulative rainfall (mm) by countries or territories. The ER relationships were fitted using TC-specific RRs estimated by excluding a 30-, 60- or 90-day post-TC period.


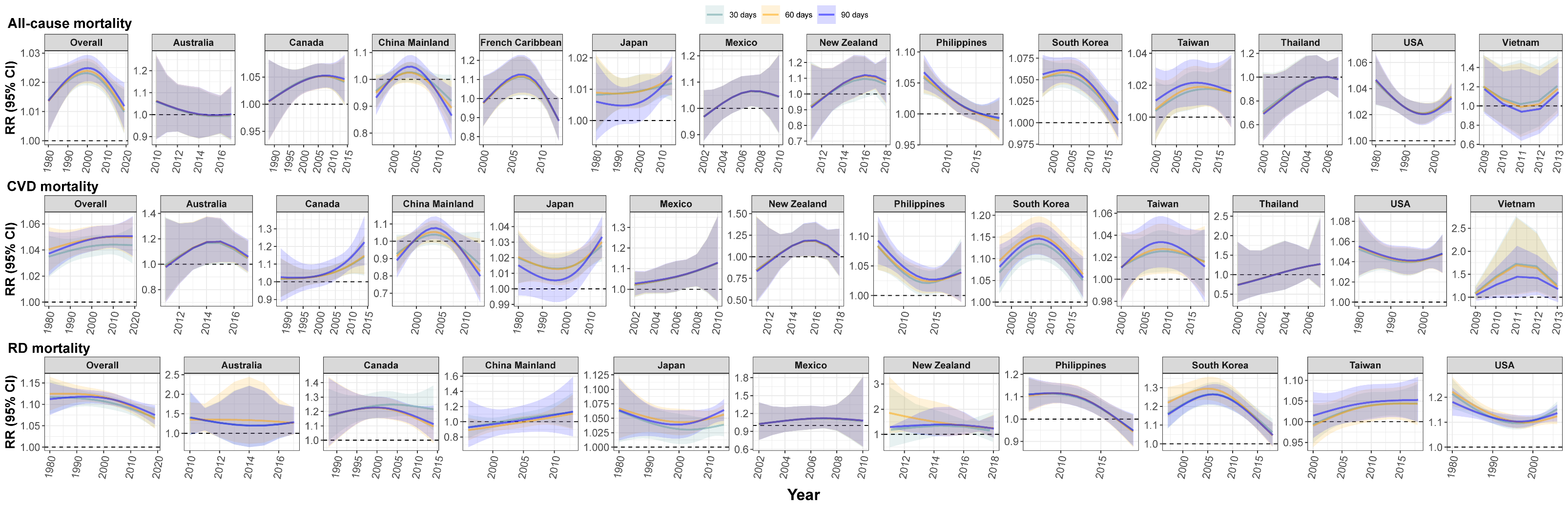


**Fig D**. The temporal trend of the relative risks (RR) for all-cause, cardiovascular diseases (CVD) and respiratory diseases (RD) mortality after TC exposure from 1980 to 2019 by countries or territories. The temporal trends were fitted using TC-specific RRs estimated by excluding a 30-, 60- or 90-day post-TC period.
